# Geographically Widespread Swordfish Barcode Stock Identification: A Case Study of Its Application

**DOI:** 10.1371/journal.pone.0025516

**Published:** 2011-10-19

**Authors:** Anna Maria Pappalardo, Francesca Guarino, Simona Reina, Angela Messina, Vito De Pinto

**Affiliations:** Section of Biochemistry and Molecular Biology, Department of Biological, Geological and Environmental Sciences, University of Catania, National Institute of Biomembranes and Biosystems, Section of Catania, Catania, Italy; Biodiversity Insitute of Ontario - University of Guelph, Canada

## Abstract

**Background:**

The swordfish (*Xiphias gladius*) is a cosmopolitan large pelagic fish inhabiting tempered and tropical waters and it is a target species for fisheries all around the world. The present study investigated the ability of COI barcoding to reliably identify swordfish and particularly specific stocks of this commercially important species.

**Methodology:**

We applied the classical DNA barcoding technology, upon a 682 bp segment of COI, and compared swordfish sequences from different geographical sources (Atlantic, Indian Oceans and Mediterranean Sea). The sequences of the 5′ hyper-variable fragment of the control region (5′dloop), were also used to validate the efficacy of COI as a stock-specific marker.

**Case Report:**

This information was successfully applied to the discrimination of unknown samples from the market, detecting in some cases mislabeled seafood products.

**Conclusions:**

The NJ distance-based phenogram (K2P model) obtained with COI sequences allowed us to correlate the swordfish haplotypes to the different geographical stocks. Similar results were obtained with 5′dloop. Our preliminary data in swordfish *Xiphias gladius* confirm that Cytochrome Oxidase I can be proposed as an efficient species-specific marker that has also the potential to assign geographical provenance. This information might speed the samples analysis in commercial application of barcoding.

## Introduction

The swordfish *Xiphias gladius* is one of the most widely distributed species of pelagic fish commonly found in the tropical and temperate zones of the Atlantic, Indian and Pacific Oceans. Its life history characteristics and its high mobility suggest a high gene flow and little genetic subdivision among individuals and pose difficulties in defining and delineating its stocks. Although the swordfish (*X. gladius*) is considered to be a highly migratory cosmopolitan fish species, conventional restriction and nucleotide sequence analyses of the mitochondrial DNA (mtDNA) have revealed the population to be structured not only between but also within ocean basins [1]–[4]. The global genetic population structure of swordfish has been partly elucidated so far. Several genetic studies have demonstrated that swordfish populations are subdivided, mainly on an ocean-basin scale, with a highly distinct stock in the Mediterranean, two stocks in the Atlantic (North and South) with disputed boundary, and an Indo-Pacific stock [1]–[3], [5]. However, there are indications for further subdivision within Indian [6] and Pacific Ocean [7]–[9]. Genetic studies conducted in recent years to examine the global population structure of swordfish have suggested some broad-scale differences. Comparison of two samples collected in the eastern and western Pacific identified significant differences at one (PROT- 3*) of four polymorphic allozyme loci but no difference in the frequency of mtDNA restriction fragment length polymorphisms (RFLP) [10], [7]. However, at least four groups (Pacific, Mediterranean, North Atlantic, South Atlantic) were indicated by mtDNA RFLP analyses [4]. Sequence analysis of the mtDNA control region have identified two major clades, with a subdivision of clade II indicating respectively monophyletic groups in the Atlantic Ocean and the Mediterranean Sea [11]. These subdivisions have been supported by RFLP and sequence analysis of nuclear genes [12], [5]. Only a limited attention has been paid to population structures within the Mediterranean Sea. RFLP analysis of mtDNA in three Mediterranean areas and the adjacent Atlantic Ocean off Gibraltar (Tarifa) revealed no heterogeneity in haplotype distribution [1]. Similarly, no differentiation has been detected between Mediterranean and Tarifa samples by variants of a nuclear gene (calmodulin) or by RFLP analysis of the mtDNA control region [5]. Pujolar et. al. [13] examined the population structure of swordfish in the Mediterranean Sea using allozyme data. The lack of temporal or spatial heterogeneity found in their study is consistent with a single population of swordfish in the Mediterranean Sea and adjacent waters of the Atlantic Ocean.These studies have resulted in different conclusions regarding the population structure of swordfish and, hence, there has been no consensus on the Mediterranean stock structure of swordfish.

*X. gladius* is a species widely commercialized in the fishing industry. In food safety and traceability, consumers are more and more demanding about composition and provenance of processed seafood products. In the trade of many species, manufacturing alterations usually bring to the loss of any morphological diagnostic features of the species, enhancing the possibility of fraudulent substitutions and incorrect product labeling. A very common fraud in Sicily is the substitution of fresh swordfish from Mediterranean area with frozen fishes, usually imported from the North Atlantic or the Indian Ocean. This fraud is particularly easy since this kind of fish is sold sliced.

DNA barcoding is a method for species identification that is based on the surveillance of sequence diversity in a 650 bp region of the mitochondrial gene coding for cytochrome c oxidase I (COI) [14]. This gene region generally shows little variation within a species but substantial divergence between species, allowing for species differentiation. In this approach for species identification, the DNA barcode of an unknown sample is screened against a reference sequence library and a species assignment is made when the query sequence matches just one of the species in the reference library. A reference library of DNA barcodes for all fish species is currently under assembly by the Fish Barcode of Life campaign (FISH-BOL) [15]. With records now in place for more than 6500 species, barcodes have proven to unambiguously discriminate about 93% of freshwater species and 98% of marine species. DNA barcode is emerging as a powerful tool for food authentication or food safety, as well as other aspects of fisheries management [16], since it is a rapid, cost-effective and broadly applicable molecular diagnostic technique. Seafood authentication and safety concerns are a growing issue in today's global marketplace, because traditional morphology-based identification keys and existing molecular approaches have limitations for species identification [17], [18]. In this work we have verified the reliability of DNA barcoding in the recognition of commercialized swordfish against possible commercial frauds in local markets. Furthermore we have tested the hypothesis of COI gene also as a suitable DNA marker for the Mediterranean stock identification.

## Results

### Analysis of COI sequences

Unambiguously aligned sequences were obtained for 682 bp of COI sequence from 65 tissue samples of *X. gladius*. All sequences were deposited in GenBank (Table S1). No insertions, deletions or stop codons were observed in any sequence. The lack of stop codons is consistent with all amplified sequences being functional mitochondrial cox1 sequences, and that, together with the fact that all amplified sequences were 682 bp in length, suggests that NUMTs (nuclear DNA sequence originating from mitochondrial DNA sequences) were not sequenced (vertebrate NUMTS are typically smaller than 600 bp [19]).

A total of 15 nucleotide sites were found variable of which 11 positions were parsimony informative (Table S2). These polymorphisms defined 16 distinct swordfish haplotypes, only 1 of which (H10) was shared among Atlantic and Indian Ocean populations. Relatively high values of haplotype diversity were found in Atlantic and Mediterranean samples (0.80 and 0.84 respectively), while the lowest value was observed in Indian sample (Table 1).

**Table 1:** Summary of the genetic diversity indexes in *Xiphias gladius* screened in this work.

		5′dloop		COI	
	*N*	*h*	*π*	*h*	*π*
Atlantic ocean	20	0.990 (0.018)	0.036 (0.019)	0.800 (0.047)	0.002 (0.001)
Mediterranean Sea	30	0.924 (0.033)	0.054 (0.027)	0.839 (0.053)	0.005 (0.003)
Indian ocean	15	0.762 (0.066)	0.017 (0.009)	0.419 (0.113)	0.001 (0.001)
Average		0.972 (0.008)	0.040 (0.003)	0.887 (0.023)	0.004 (0.0004)

*N*: number of *Xiphias gladius* screened per sampling site; *h*: haplotype diversity; *π*: nucleotide diversity.

Standard deviation in parentheses.

### Molecular features of the mitochondrial control region (5′dloop)

A sequence of 413 bp in the control region was used for the following analyses. The nucleotide composition of the swordfish control region was AT-rich (64%). A total of 97 polymorphic sites, including 23 singletons and 74 parsimoniously informative sites, were identified within this stretch of sequence (Table S3), resulting in 36 distinct haplotypes among the 65 individuals. All sequences were deposited in GenBank (Tab. S4).

Notably, there were 14 unique haplotypes discoveredamong 30 individuals of swordfish collected from the Mediterranean Sea; 17 unique haplotypesamong 20 individuals from the Atlantic Ocean; 3 unique haplotypes from the Indian Ocean samples and 2 shared haplotypes (1 between Atlantic Ocean and Mediterranean Sea, 1 between Atlantic and Indian Ocean). As expected, the genetic diversity revealed by mitochondrial control region was much higher than for COI swordfish sequences. Very large values of haplotypic diversity *h* [20] were estimated for all populations. The haplotypic diversity for the Atlantic sample was 0.99. Lower diversity values were obtained for the Mediterranean and Indian, but the values for these regions were still high (0.92 and 0.76, respectively) (Table 1).

### TACA sequence repeats

**Figure 1:**
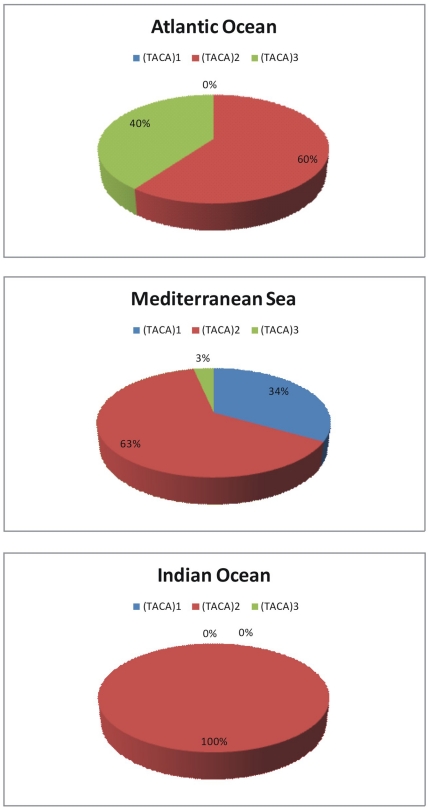
Frequency distribution of TACA repeats found in this study.

One, two or three contiguous 5′-TACA-3′ sequence repeats were found at the 5′-end of the control region in the swordfish specimens examined in this work. TACA repeats were previously found in swordfish populations of the Atlantic Ocean and Mediterranean Sea [11], [1]. In this study, only the Mediterranean samples had a single 5′-TACA-3′ sequence. Samples from the Oceans and from the Mediterranean Sea had two 5′-TACA-3′ repeats, and, at the end, only one samples from the Mediterranean Sea and some from Atlantic Ocean had three 5′-TACA-3′ repeats. The proportion of samples with 2 repeats from the Atlantic Ocean and from the Mediterranean Sea was larger than that with 3 repeats (Fig. 1). Previous studies [11], [2] also indicated that no single repeat sample from swordfish was discovered in the Pacific Ocean, and only a very few swordfish carrying the single 5′-TACA-3′ sequence were also discovered off western Australian waters.

### Cluster analysis

**Figure 2:**
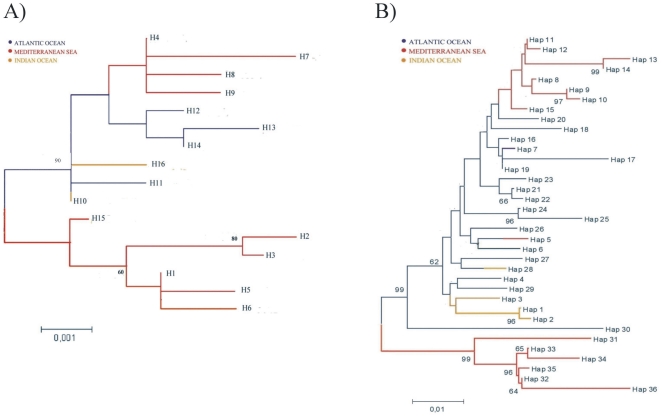
Neighbour-Joining distance-based phenograms of (a) swordfish COI barcode and (b) 5′dloop haplotypes from Mediterranean Sea, Atlantic and Indian Oceans identified in this study. Bootstrap values greater than 50 are shown (1000 replicates).

The unrooted neighbor-joining phenogram in Figure 2a clearly shows the relationships between all COI haplotypes. In particular, there are two main clusters, one of which includes only haplotypes Mediterranean. In the second cluster there are more different haplotypes. A second node separates four Mediterranean haplotypes (H4, H7, H8, H9) from the Atlantic ones (H12, H13, H14) and within this second cluster there are Atlantic haplotypes mixed with Indian ones. The NJ phenogram (Figure 2b) inferred from 5′dloop haplotypes revealed awell-supported cluster(99%) exclusively containing Mediterranean haplotypes, while other Mediterranean haplotypes are arranged on the other side of the tree, and are muchcloser tothe Atlantic haplotypes. It is likely that these twoclades correspond to those previously identified by Alvarado Bremer et al. [11], [3] using both parsimony and neighbour-joining analyses. Moreover, clade II swordfishes are distinguished by a single repeat of the motif TACA at the beginning of the fragment, whereas clade I sworfishes have two or three contiguous repeats of the motif at this position [11]. In agreement with a previous study [21], clade I was subsequently subdivided into a group presenting two TACA repeats and another with three TACA repeats.

**Figure 3:**
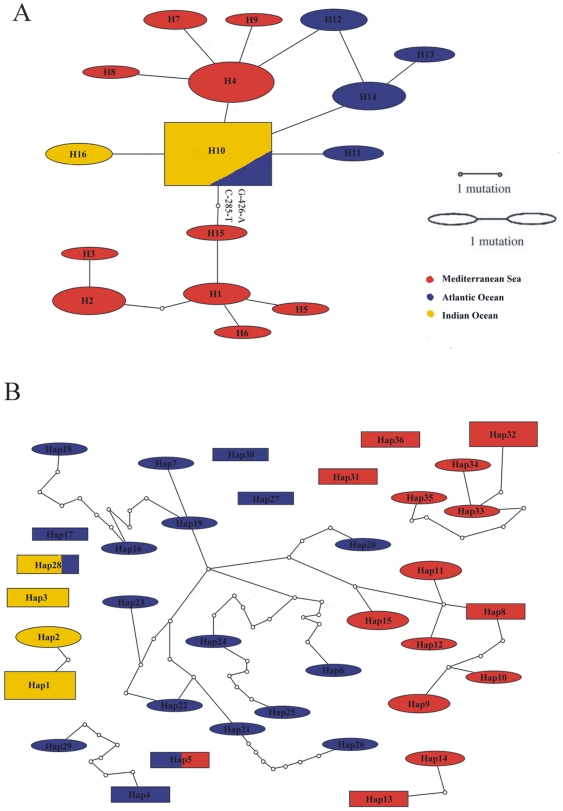
Parsimony network analysis of (A) the 16 COI swordfish haplotypes and (B) the 36 5′dloop haplotypes. Each connection represents one mutational step; small circles represent unsampled haplotypes. The size of each haplotype node is proportional to the number of specimens representing each haplotype. The total number of specimens in this network is 65. The network was generated using TCS 1.21 (available at http://darwin.uvigo.es/software/tcs.html) at the default 95% connection limit.

The NJ phenograms were supported by the parsimony network analyses that produced one haplotype group from COI sequences (Fig. 3A) and several for the 5′dloop sequences (Fig. 3B). In particular, Fig. 3A shows that H10 is the most common haplotype, shared between Indian and Atlantic Oceans. Mediterranean haplotypes H1, H2, H3, H5, H6, H15 are separated from H10 by two mutational steps (G-426-A and C-285-T) that represent their nucleotide diagnostics (ND) [22]. Another group of Mediterranean haplotypes (H4, H7, H8, H9) is connected with the Atlantic haplotype H12 and with the H10, that appears as most common haplotype.

In Fig. 3B the parsimony network includes a much larger number of haplotypes, due to the higher polymorphism of the 5′dloop sequences. The Mediterranean haplotypes Hap13–14, Hap 32–35, Hap 36, Hap 31 are organized in four separated groups. Other Mediterranean haplotypes Hap8–12, Hap 15 are connected to Atlantic haplotypes but through several (5) mutational steps. Interestingly this analysis showed that Indian Ocean haplotypes are also separated from the others with the exception of Hap28, shared by Indian and Atlantic samples.

### Screening of unknown samples: a case-report of fraudolent substitution

**Figure 4:**
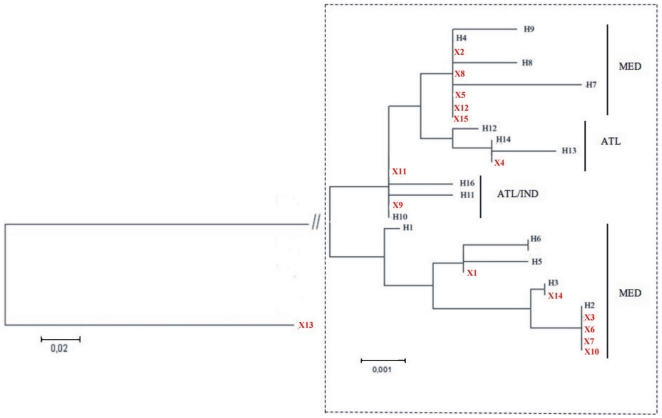
Neighbour-Joining distance-based phenogram (K2P) between sequences of COI in unknown origin samples (but all of them labeled as Mediterranean) purchased at local supermarkets. The branch connecting the X13 (outgroup) sample to the ingroup was re-scaled in order to focus on the differences within *X. gladius*.

**Figure 5:**
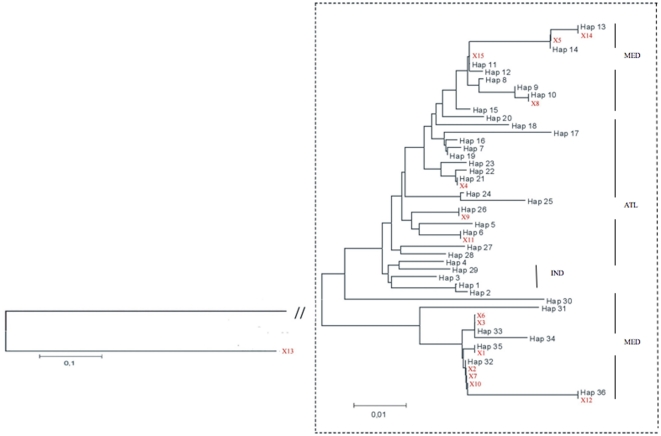
Neighbour-Joining distance-based phenogram (K2P) between sequences of 5′dloop in unknown origin samples (but all of them labeled as Mediterranean) purchased at local supermarkets. The branch connecting the X13 (outgroup) sample to the ingroup was re-scaled in order to focus on the differences within *X. gladius*.

Samples of sliced swordfishes were obtained from the local market and processed to sequence the COI and 5′dloop regions. All these samples were claimed to be fishes from the Mediterrean Sea. The sequences obtained were included into the previous analyses and new NJ trees were built, with the aim to assign the correct provenance to the unknown samples. Interestingly, the sample named X13 was located outside the trees. The identification of the sequence of X13 revealed that it was a shark, *Prionace glauca* (Figures 4–5). Three out of 14 remaining unknown samples were found to cluster with Atlantic Ocean with both markers (samples X4, X9, X11) and only 11 samples clustered with the fishes from Mediterranean Sea, as it was originally declared by the sellers (Figures 4–5). In particular, in Table 2 we can observe that there is a strong agreement between both markers (COI and 5′dloop) and all the Mediterranean specimens, with the exception of X12, can be assigned at the same phylogenetic clade (Clade I or Clade II as defined in [11], [3]).

**Table 2:** Assignment of unknown samples to capture regions.

	REGION OF CAPTURE	
N	COI	DLOOP
x1	ME (II)	ME (II)
x2	ME (I)	ME (I)
x3	ME (II)	ME (II)
x4	ATL	ATL
x5	ME (I)	ME (I)
x6	ME (II)	ME (II)
x7	ME (II)	ME (II)
x8	ME (I)	ME (I)
x9	ATL	ATL
x10	ME (II)	ME (II)
x11	ATL	ATL
**x12**	**ME (I)**	**ME (II)**
x13	*Prionace glauca*	*Prionace glauca*
x14	ME (I)	ME (I)
x15	ME (I)	ME (I)

The numbers in brackets refers to the two different phylogenetic clades detected in our work and supported by [11], [3].

In bold a sample whose clades'assignment is ambiguous.

## Discussion

There is a wide array of molecular methodologies currently available for species identification [23]. More recently, ‘DNA barcoding’, the survey of sequence diversity in a 648 bp segment of the mtDNA gene cytochrome c oxidase subunit I (COI), has been proposed as a standard tool for species-level identifications of all animals. Many studies have shown its effectiveness for species identification in various animal lineages [14], [23]–[30] including fishes [31], [32]. Ward et al. [31] provided early evidence for the efficacy of DNA barcoding in marine fish identification in a study that examined more than 200 Australian species. In general, COI-DNAbarcoding has proven to be an efficient tool to identify samples of unknown origin and then to control the information providedon product labels. On the other side, to characterize fish stocks, markers with a higher rate of intraspecific variability are generally used, such as the left domain of mitochondrial control region (5′dloop). This usually is the fastest evolving region in the mitochondrial DNA of vertebrates and invertebrates, and therefore it is more sensitive than protein loci as a marker of phylogeographic structuring of many organisms [33], [34].

TheestimatefortheCOIhaplotypediversity average (*h* = 0.89) is highbutnotas high asthat for 5′dloop (*h* = 0.97). Both gene regions have shownasimilarpatternof intraspecific relationships butwith the lowestestimateofhaplotypediversity the COI sequences are less at risk of mutational saturation than the dloop sequences.

In this study we have used the COI sequence to get species identity of swordfish samples and define whether such marker is also informative about the geographical stock of origin. For comparison, the 5′ fragment of the control region (5′dloop) of the same samples was also sequenced. The samples analyzed were obtained by official fishery enterprises, under a veterinary control, and were origin-certified from Atlantic and Indian Ocean and from the Mediterranean Sea. The sequencing work allowed us to draw a reference picture for the species.

The presence of polymorphisms in COI alignments allowed us to define 16 distinct swordfish haplotypes, only 1 of which (H10) was shared among Atlantic (35%) and Indian (65%) Ocean populations. Relatively high values of haplotype diversity were found in Atlantic and Mediterranean samples (*h* = 0.80 and *h* = 0.84 respectively), while the lowest value was observed in Indian sample. NJ distance-based phenograms showed the presence of a well separated Mediterranean haplotype cluster, whose nucleotide diagnostics (ND) have been identified [22]. In the Mediterranean samples we also visualized another cluster more closely connected with some Atlantic haplotypes. These data were supported by the parsimony network analysis.

From the analysis of the control region, the highest values of haplotype diversity was recorded in samples from the Atlantic Ocean (*h* = 0.99), as reported by Alvarado Bremer et al. [3], and a lower value was obtained for the Mediterranean (*h* = 0. 92). Indeed, the analysis of COI sequences revealed that the widest haplotype and nucleotide diversity was found in the specimens from the Mediterranean Sea. NJ distance-based phenogram of swordfish control region revealed the presence of two different and well supported (bootstrap value = 0.99%) clades. We can assume that these clades correspond to those (Clade I and Clade II) reported in previous population genetic studies as single TACA repeat was present exclusively in Mediterranean samples but not in the oceans samples [3], [9], [11]. TACA repeats have been specifically found in the 5′dloop of *X. gladias* [11], [1]. In fact, our NJ phenogram results revealed that Clade I includes 30 haplotypes corresponding to individuals that were collected in all the ocean basins sampled. Conversely, Clade II includes 6 haplotypes, numbered 31–36 in this work. Members of Clade II were found exclusively in the Mediterranean Sea.

### Is the Mediterranean lineage a differentiated intraspecific stock or is it a candidate cryptic species?

The swordfish structure appears quite important with populations subdivided in oceanic and infra-oceanic scales, in the Atlantic [3], [9] and Pacific oceans [6], [8] and in the Mediterranean Sea [1]. Mitochondrial DNA analyses by different authors [35], [1]–[4] have revealed at least four breeding units (stocks) of the swordfish: Mediterranean, North Atlantic, South Atlantic and Indo-Pacific. Some corridors seem to exist between oceans but these appear to be constrained and delineated by equatorial boundaries. For example, swordfish appear quite similar between South-Indian and South-Atlantic oceans, as well as between South Indian and Pacific oceans [5], [6], more similar between neighbouring oceans than from the southern to the northern parts of the same ocean. While most of the genetic studies on the swordfish performed until now have involved only one genetic marker, conclusions based both on nuclear and mitochondrial DNA seem to agree in highlighting differences between the Atlantic Ocean and the Mediterranean Sea [12]. Atlantic and Mediterranean swordfish are differentiated due to the combined effects of vicariance, secondary contact, and dissimilar regional demographic histories. In addition, the allozymic analysis of Mediterranean swordfish population described low genetic distances between Mediterranean and Atlantic stocks, while revealing a definite and meaningful genetic separation between swordfish stocks from Eastern or Western areas of the Mediterranean Sea [13].

By means of COI and a 5′dloop sequences, we have shown that two mtDNA *Xiphias gladius* phylogenetic lineages exist. Moreover,our evidence suggests that the COI barcode is not only an efficient species-specific marker, but, at least for the swordfish, it also has been probed to discriminate between Mediterranean haplotypes and those from Oceans.

These results, compared with data from the literature [12], [13], [35], [36] would seem to suggest an alternative explanation for the clear structuration of the Mediterranean swordfish samples. A possible explanation would be that *X. gladius* Mediterranean distinct lineage could be a candidate cryptic species (sensu Padial et al. [37]) distinct from the more broad-ranging species which is shown to have some population level structuring relevant to Ocean basins. This organization would justify the detection of a localized stock with a barcode-based approach, while some level of phylogeographic assessment could be attributed with barcodes to the other, more broad-ranging species.

Further analysis with a larger sample size is necessary to get final conclusions about this hypothesis.

### A market case identification report: is COI useful as a stock attribute in addition to be a species identificator?

This research was also prompted by the practical need of recognizing swordfish caught in the Mediterranean, and sold as fresh one, from the same fish caught in the Atlantic and Indian Oceans, kept frozen but later sold as fresh, provoking an important commercial fraud.

We thought that eventually the “classical” barcode could be informative enough not only to distinguish among species but also to shed some light into the geographical stocks of the same species. In this work we sequenced additional samples from the food market and we could confirm the assignment of geographical origin of the unknown swordfish samples only in 11 cases. Three samples have been assigned to FAO regions different from those claimed and one was from a different species (*Prionace glauca*), thus a genuine commercial fraud. In addition we could verify that COI barcode and 5′dloop gave overlapping information.

This means than, upon species validation, it is possible to approximately confirm a geographical origin with a single sequencing analysis. In specific, validated situations, the possibility to have in only one run both the species identification and the stock discrimination would be a big enhancement in the practical analysis.

In conclusion, by means of COI and a 5′dloop sequences we have shown that two mtDNA *Xiphias gladius* phylogenetic lineages exist. Moreover,our evidence suggests that the COI barcode is not only an efficient species-specific marker, but, at least for the swordfish, it also could be considered to discriminate between Mediterranean haplotypes and those from Oceans. The practical consequence is that it is possible to utilize the COI barcode as an useful tool not only to identify species but, when validated and in a limited extent, to identify the geographical origin.

## Materials and Methods

### Samples and DNA extraction

We obtained a total of 65samples of *X. gladius* from import/export companies that could certify the fish origin. Samples from Indian (N = 15) and Atlantic (N = 20) Oceans and Mediterranean Sea (N = 30) were labeled after arriving at the laboratory and preserved at −80°C until DNA extraction. In addition 15 fish slices, claimed to be fresh swordfish of Mediterranean origin, were randomly collected from local supermarkets and were used as “unknown” samples (Table 3). Total genomic DNA was extracted using DNeasy Tissue Kit (Qiagen) following the manufacturers instructions.

**Table 3:** Collection information of samples used in this study.

Region	Sampling date	N	FAO zone
Mediterranean Sea	May 08, June 09	30	FAO 37
Atlantic Ocean	June 09	20	FAO27
Indian Ocean	May–June 09	15	FAO 51
“Unknown samples”	May–July 10	15	unknown

“Unknown samples” are fresh or frozen sliced specimens bought at local fishmarkets.

All of them were labeled as FAO37 zone origin.

### PCR amplification and sequencing of mtDNA cytochrome oxidase I (COI) and mtDNA control region

Cytochrome oxidase I sequences were obtained usingthe primer combination of universal primers VF2_t1- 5′ TGTAAAACGACGGCCAGTCAACCAACCACAAAGACATTGGCAC-3′ and FishR2_t1-5′CAGGAAACAGCTATGACACTTCAGGGTGACCGAAGAATCAGAA-3′ described in Ward et al. [31]. The amplification was carried out in 25 µl using approximately 50 ng of the isolated DNA as a template. In addition, each PCR reaction contained 1× Taq DNA polymerase buffer (supplied by the respective Taq DNA polymerase manufacturer), 1.5–2 mM of MgCl2, 200 mM of each dNTP, 10 pmols of each primer and 0.5 U of Taq DNA polymerase (Platinum Taq DNA polymerase, Invitrogen). Thermal cycles involved an initial denaturing step of 2 min at 94°C, followed by 35 cycles of denaturation at 94°C for 30 s, annealing at 52°C for 45 s and extension at 72°C for 1 min. Negative controls were included in all PCR runs to ascertain that no cross-contamination took place. Double-stranded products were checked in agarose gel electrophoresis and purified with the Qiaquick PCR purification kit (Qiagen) and subsequently sequenced in the forward and reverse direction. For the mtDNA 5′dloop a sequence of 413 bp (longer than used in previous swordfish studies [3], [35]) of the first (left) domain of the mitochondrial control region was obtained using the primer combination of L15998 and H235 [3] with the same PCR profiles. Sequencing was performed on both strands using an ABI 137Prism 3100 automated sequencer (Applied Biosystems). Sequences have been carefully checked and deposited to Genbank (Tables S1 and S2).

### Identification of unknown samples sold as “swordfish” and estimation of commercial frauds

The identification of unknown samples was conducted with the Identification Engine tool (IDS) at BOLD (searching on the Reference Barcode Database and considering only matches up to 98% of specimen similarity). The highest percentage of similarity obtained with this approach was compared to the labeled name recorded at the market in order to determine the percentage of species substitution.

### Sequence analysis

For each genetic marker the sequences obtained were aligned using ClustalX [38]. Ambiguous regions of the alignment were systematically identified and removed using the programme GBlocks [39]. The default program parameters were used, with the exclusion of a minimum block length of 5 and gaps in 50% of positions. Sequences were run through Collapse 1.2 (available at http://darwin.uvigo.es) in order to distill the sequences into unique haplotypes. The barcode sequences were used to determine two parameters: nucleotide diversity (π) described as the mean number of pairwise differences, and gene diversity (*h*), calculated using DnaSP ver. 3 [40]. These parameters were indeed used to detect intraspecific genetic variability. In order to identify nucleotide diagnostics (NDs) [22] for “a Mediterranean lineage” we used MEGA version 4.0 [41] to display the aligned sequence data and highlight all variable sites. NDs for a target species were easy to identify with a visual scan when the “use identical symbol” option was enable in MEGA.Relationships among the sequence haplotypes obtained were examined using Neighbour-Joining (NJ) method. The NJ phenogram was constructed using pairwise distances calculated following the application of Kimura's two-parameter (K2P) correction for multiple substitutions in MEGA version 4.0 [41]. No outgroup was employed to root the gene-tree because of the extreme distances separating this species from istiophorid species. The robustness of internal branches of distance was estimated by bootstrapping [42] with 1000 replicates.

Moreover, the relationships between unique COI and control region haplotypes were described with a parsimony network generated by the program TCS ver. 1.21 [43].

## Supporting Information

Table S1 List of CoI haplotypes.
(DOC)

Table S2 Variable nucleotide sites from 682 bp sequences of the partial mitochondrial cytochrome oxidase I in swordfish haplotypes.
(DOC)

Table S3 Variable nucleotide sites in the 43 bp sequences of the swordfish 5′dloop examinated in this work.
(DOC)

Table S4 List of dloop haplotypes.
(XLS)
