# Supplementary material for: Geographically Widespread Swordfish Barcode Stock Identification: A Case Study of Its Application
Source: PLoS One. 2011 Oct 19;6(10):e25516. doi: 10.1371/journal.pone.0025516 (PMC3198442; doi:10.1371/journal.pone.0025516)
Supplement: Table S2 — Variable nucleotide sites from 682 bp sequences of the partial mitochondrial cytochrome oxidase I in swordfish haplotypes. (DOC) [file pone.0025516.s002.doc]

**Table S2 - Variable nucleotide sites from 682 bp sequences of the partial mitochondrial cytochrome oxidase I in swordfish haplotypes.**

| **Haplotype** | **Nucleotide position** | | | | | | | | | | | | | | |
| --- | --- | --- | --- | --- | --- | --- | --- | --- | --- | --- | --- | --- | --- | --- | --- |
|  | 8 | 24 | 43 | 105 | 116 | 174 | 213 | 270 | 285 | 426 | 483 | 504 | 564 | 648 | 676 |
| HAP1 | T | A | G | G | T | T | A | A | T | A | G | G | G | C | G |
| HAP2 | G | A | C | G | T | T | A | A | T | A | G | G | G | C | G |
| HAP3 | G | A | C | G | T | T | A | A | C | A | G | G | G | C | G |
| HAP4 | T | A | G | G | T | C | A | A | C | G | G | G | A | C | G |
| HAP5 | T | A | G | G | T | T | A | A | T | A | G | G | G | T | G |
| HAP6 | T | A | G | G | T | T | A | A | T | A | G | G | G | C | A |
| HAP7 | T | A | G | T | T | C | A | A | C | G | G | G | A | C | G |
| HAP8 | T | A | G | G | T | C | A | C | C | G | G | G | A | C | G |
| HAP9 | T | A | G | G | A | C | A | A | C | G | G | G | A | C | G |
| HAP10 | T | A | G | G | T | T | A | A | C | G | G | G | A | C | G |
| HAP11 | T | A | G | G | T | T | T | A | C | G | G | G | A | C | G |
| HAP12 | T | G | G | G | T | C | A | A | C | G | G | G | A | C | G |
| HAP13 | T | G | G | G | T | T | A | A | C | G | A | G | A | C | G |
| HAP14 | T | G | G | G | T | T | A | A | C | G | G | G | A | C | G |
| HAP15 | T | A | G | G | T | T | A | A | T | A | G | G | A | C | G |
| HAP16 | T | A | G | G | T | T | A | A | C | G | G | A | A | C | G |
