# Supplementary material for: Geographically Widespread Swordfish Barcode Stock Identification: A Case Study of Its Application
Source: PLoS One. 2011 Oct 19;6(10):e25516. doi: 10.1371/journal.pone.0025516 (PMC3198442; doi:10.1371/journal.pone.0025516)
Supplement: Table S3 — Variable nucleotide sites in the 43 bp sequences of the swordfish 5′dloop examinated in this work. (DOC) [file pone.0025516.s003.doc]

Table S 3 – Variable nucleotide sites in the 43 bp sequences of the swordfish 5’dloop examinated in this work
