# Supplementary material for: Geographically Widespread Swordfish Barcode Stock Identification: A Case Study of Its Application
Source: PLoS One. 2011 Oct 19;6(10):e25516. doi: 10.1371/journal.pone.0025516 (PMC3198442; doi:10.1371/journal.pone.0025516)
Supplement: Supplementary file 3 [file pone.0025516.s001.doc]

Table S1 - List of COI haplotypes.

COI Haplotype	N	Capture region	Accession number
H1	4	MED FAO37	JN049558
H2	6	MED FAO37	JN049559
H3	1	MED FAO37	JN083386
H4	10	MED FAO37	JN083387
H5	1	MED FAO37	JN083388
H6	1	MED FAO37	JN083389
H7	3	MED FAO37	JN083390
H8	1	MED FAO37	JN083391
H9	1	MED FAO37	JN083392
H10	6+11	ATL FAO27/IND FAO51	JN083393
H11	2	ATL FAO27	JN083394
H12	4	ATL FAO27	JN083395
H13	2	ATL FAO27	JN083396
H14	6	ATL FAO27	JN083397
H15	2	MED FAO37	JN083398
H16	4	IND FAO51	JN083399

N = number of specimens.
